# Rapid Segmentation and Diagnosis of Breast Tumor Ultrasound Images at the Sonographer Level Using Deep Learning

**DOI:** 10.3390/bioengineering10101220

**Published:** 2023-10-19

**Authors:** Lei Yang, Baichuan Zhang, Fei Ren, Jianwen Gu, Jiao Gao, Jihua Wu, Dan Li, Huaping Jia, Guangling Li, Jing Zong, Jing Zhang, Xiaoman Yang, Xueyuan Zhang, Baolin Du, Xiaowen Wang, Na Li

**Affiliations:** 1 Strategic Support Force Medical Center, Beijing 100024, China; leiyang1105@163.com (L.Y.); jh_02821@126.com (J.W.); 13388715773@163.com (J.Z.);; 2 Chongqing Zhijian Life Technology Co., Ltd., Chongqing 400039, China; 15755082328@163.com (B.Z.);; 3 State Key Laboratory of Processors, Institute of Computing Technology, Chinese Academy of Sciences, Beijing 100049, China; renfei@ict.ac.cn; 4 Central Medical District of Chinese PLA General Hospital, Beijing 100080, China

**Keywords:** convolutional neural network, ultrasound, breast cancer, tumor identification, auxiliary diagnosis

## Abstract

Background: Breast cancer is one of the most common malignant tumors in women. A noninvasive ultrasound examination can identify mammary-gland-related diseases and is well tolerated by dense breast, making it a preferred method for breast cancer screening and of significant clinical value. However, the diagnosis of breast nodules or masses via ultrasound is performed by a doctor in real time, which is time-consuming and subjective. Junior doctors are prone to missed diagnoses, especially in remote areas or grass-roots hospitals, due to limited medical resources and other factors, which bring great risks to a patient’s health. Therefore, there is an urgent need to develop fast and accurate ultrasound image analysis algorithms to assist diagnoses. Methods: We propose a breast ultrasound image-based assisted-diagnosis method based on convolutional neural networks, which can effectively improve the diagnostic speed and the early screening rate of breast cancer. Our method consists of two stages: tumor recognition and tumor classification. (1) Attention-based semantic segmentation is used to identify the location and size of the tumor; (2) the identified nodules are cropped to construct a training dataset. Then, a convolutional neural network for the diagnosis of benign and malignant breast nodules is trained on this dataset. We collected 2057 images from 1131 patients as the training and validation dataset, and 100 images of the patients with accurate pathological criteria were used as the test dataset. Results: The experimental results based on this dataset show that the MIoU of tumor location recognition is 0.89 and the average accuracy of benign and malignant diagnoses is 97%. The diagnosis performance of the developed diagnostic system is basically consistent with that of senior doctors and is superior to that of junior doctors. In addition, we can provide the doctor with a preliminary diagnosis so that it can be diagnosed quickly. Conclusion: Our proposed method can effectively improve diagnostic speed and the early screening rate of breast cancer. The system provides a valuable aid for the ultrasonic diagnosis of breast cancer.

## 1. Introduction

The American Cancer Society’s journal, CA Cancer J Clin, published [1] cancer statistics in the United States for 2023, and the report states that breast cancer alone accounts for 31 percent of cancers in women. According to the IARC statistics [2], there are 420,000 new cases of female breast cancer in China, which is one of the countries with the fastest growth rate of breast cancer incidence, exceeding the global rate of 2% per year. The incidence rate of breast cancer in rural and remote areas has increased significantly, with an annual growth rate of 6.3%. Breast cancer has become the most common malignant tumor threatening women’s health. The analysis of many prospective clinical trials of RCT [3,4,5] shows that strong and effective breast cancer screening can increase the rate of early diagnosis [6], increase the chance of a cure and reduce mortality. Breast cancer screening [7] includes an X-ray of the breast, imaging, molybdenum target, ultrasound and other means. Ultrasound, when used in the detection of human soft tissue, has a good resolution [8,9,10]; in clinical medicine, it has a good performance in identifying tiny pathological changes in the tissue. Experienced doctors can determine pathological changes via ultrasound images, which can distinguish between benign and malignant lesions [11], gaining valuable treatment time through the early screening of patients.

At present, ultrasound diagnosis has certain limitations [12]. Different hospitals have different ultrasonic machines, and the quality of images collected by using different machines differs, resulting in large differences in the characteristics expressed by the images, which will cause difficulties for doctors in diagnoses. Due to the different methods of image acquisition used by doctors with different levels of experience, a full picture of the lesion area may not be captured from multiple angles, resulting in deformations in the mass. The recognition of ultrasound greatly depends on the personal experience of the attending physician, and doctors with less experience may not be able to see the lesion area from an ultrasound image. This can result in missed diagnoses and misdiagnosis, delaying the golden time of patient treatment. In order to avoid the above limitations as much as possible, the use of computers to learn from ultrasound images [13,14,15,16], simulate the diagnosis of doctors with high experience and identify the region and category of breast tumors can greatly shorten the diagnosis time and provide doctors with an auxiliary basis for diagnoses.

In recent years, big data has helped solve the problem of feature acquisition in many image tasks, and its advantages can be increased by combining artificial intelligence technology in medical image tasks [17,18,19]. Some related work on the detection of breast tumors has used relevant methods. For example, Chen [20] et al. proposed a machine learning method that can automatically segment ultrasound tumors using the threshold features of local regions and morphological algorithms to achieve tumor localization. However, the ultrasonic image has an uneven gray scale, heavy artifacts and strong noise, and the recognition effect of tumors against a complex background is not good. Wang et al. [21] proposed a multi-view convolutional neural network based on Inception-V3 and trained small datasets using transfer learning. Du et al. [22] used an edge-enhanced anisotropic diffusion (EEAD) model to achieve data preprocessing, establish shape sample constraint terms and improve the recognition accuracy of difficult samples, with a final accuracy of 92.58%. However, the actual collection of breast ultrasound images is complicated and changeable, and there are certain limitations. Yang et al. [23] used dynamic enhanced magnetic resonance imaging (DCE-MRI) technology to propose a hybrid integrated model, MEI-CNN, at the diagnostic stage, which classified benign and malignant breast tumors through modular classification. This has certain advantages compared with a single classifier. Yu et al. [24] studied a deep learning automatic classification algorithm, TDS-Net, based on breast ultrasound images, designed a double-branch structure to enhance its ability to extract features and conducted experiments with data provided by Yunnan Cancer Hospital. The results showed that the accuracy rate was 94.67%, but the generalization performance needed to be improved.

Previous studies mostly focused on a single task and did not integrate the identification and classification of breast tumors [25,26]. The simple task of ultrasound image segmentation and classification often cannot help clinicians make a rapid diagnosis, and the actual use process will be restricted by certain conditions. Therefore, according to the needs of clinical diagnosis and treatment and the advantages of the deep learning algorithm, we proposed combining the two tasks, which not only outline the shape of the tumor, but can also be used to provide preliminary diagnostic results to form a systematic diagnosis and treatment plan [27,28] and provide a practical and effective means of auxiliary diagnostics for clinicians.

Therefore, the main contributions of our work are as follows:

- We introduce a two-stage convolutional neural network framework that integrates deep-learning-based image segmentation and classification tasks, thereby reducing the time to diagnosis and improving recognition accuracy.
- With the integration of the attention mechanism in the tumor recognition method, the breast tumor region can be located and information regarding the size and shape of its main region can be obtained. The influence of the ultrasonic background on the classification can be reduced, and the diagnostic effect of senior ultrasound doctors can be achieved.
- We validate the efficiency of the proposed method on different datasets.
- We develop an intelligent auxiliary diagnosis system for breast ultrasound images, which can realize end-to-end recognition, provide auxiliary diagnosis schemes for ultrasound doctors and improve diagnosis efficiency.

## 2. Materials and Methods

### 2.1. Breast Ultrasound Image Datasets Are Used for the Development of Multiple Convolutional Neural Networks

**Figure 1:**
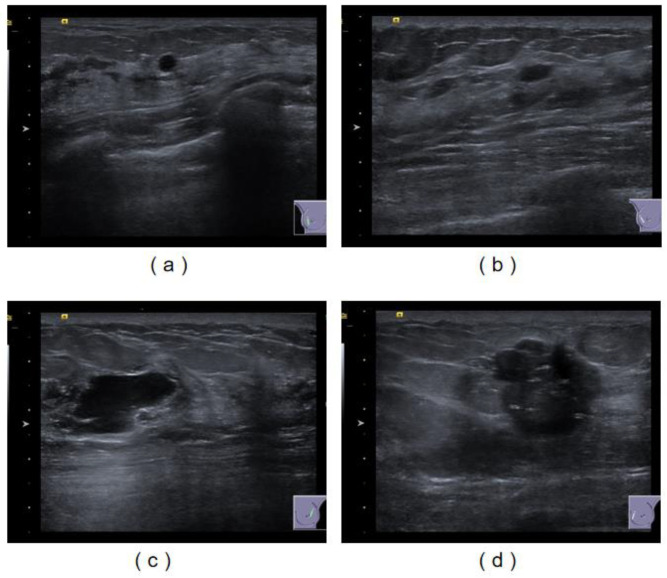
Ultrasound image of breast tumor. Among them, (**a**,**b**) are benign tumors, while (**c**,**d**) are malignant tumors.

In this study, we collected 2057 images from 1131 patients who underwent breast ultrasonography at the Strategic Support Force Medical Center in 2022. In order to avoid the influence of racial differences, the images we retained were all from female patients in China and contained at least one tumor area. For multiple images of the same patient, we used the same training set or validation set. The training set and the verification set were divided according to 8:2. All the data were used for the localization and identification of breast tumors after desensitization and for the training and verification of the classification of benign and malignant tumors. The collected data are shown in Figure 1.

After the pathological diagnosis, the images of axillary lymph node metastasis were excluded, and 264 ultrasound images of the other 100 patients were used to test the network performance, of which 56 were benign and 44 were malignant. The specific dataset distribution is shown in Table 1.

### 2.2. Data Processing

In order to train the breast tumor recognition module, we invited an experienced ultrasound physician to mark the location of the tumor and used labelme software Release 3.0 to finetune the location of the nodule. The markings cover all the outer edges of the tumor. The classification of benign and malignant tumors was also provided by a senior ultrasound doctor who had more than 20 years of experience in ultrasound diagnosis.

### 2.3. Method Overview

**Figure 2:**
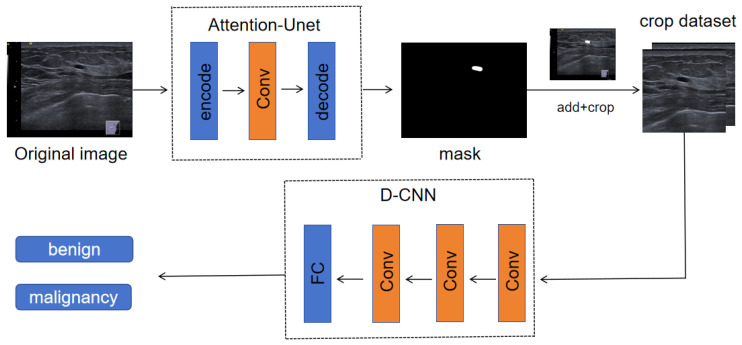
A schematic of our method. We trained a multi-convolutional neural network model, including an Attention-Unet network for region of interest identification and a D-CNN network for benign and malignant classification. The primary goal of the first-stage network is to accurately locate the breast tumor. In order to mitigate the impact of the background noise of ultrasound images, the output of the first-stage network is used as the input of the second-stage network after a post-processing step, which involves extracting image blocks from the central mask region. After that, the second-stage network, D-CNN, was used as the classification network to distinguish the tumor categories.

At present, CNNs [29] and Transformers [30] are the most widely used deep learning techniques in the field of image processing. However, the transformer-based method requires a large amount of data for training, and our data volume is small, so using CNN will not lead to overfitting on small datasets. The main structure of the CNN consists of a convolutional layer, pooling layer and fully connected layer. By fusing the above network layers, we build a fully convolutional neural network model, Attention-Unet [31], to identify the location of breast tumors from end to end and then use D-CNN, another network classifier based on deep learning, to classify the tumors. The overall flow chart is shown in Figure 2.

**Figure 3:**
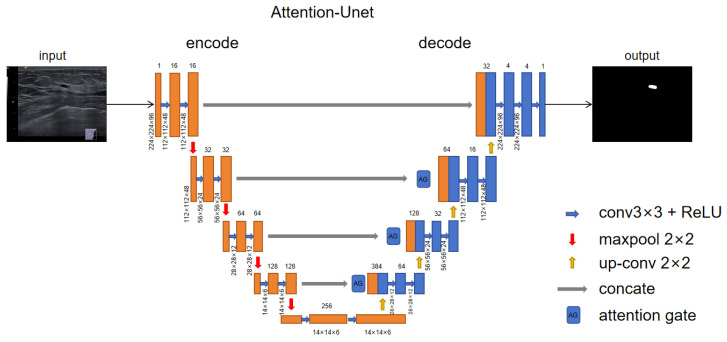
Attention-Unet network structure. We used the Unet+Attention module as the feature extractor of the first-stage network, the main purpose of which was to improve the weight of position information by fusing multi-layer feature maps and to perform end-to-end output through an encoding layer and decoding layer.

In the first stage of the network, all the input images are fed, together with their masks, into the Attention-Unet network, which contains both the encoding and decoding modules. First of all, the input image enters the coding module; the coding part is taken by using the convolutional layer as the feature extraction layer; and then the pooling layer is used to reduce the size of the feature map to obtain high-level semantic features. The decoding part integrates the output feature map after encoding, combined with a series of upsampling operations, and finally outputs the original size feature map. In feature maps of different sizes, the location information of breast tumors is different, and it is easy to lose the location information of tumors during the network learning process, resulting in the misidentification of non-tumor regions and strong similarity boundaries. Therefore, we added the attention module to the network layer to selectively learn the input features, and we added fusion operations to the upsampling to fuse the feature map information of different scales so that the network could learn the features of tumor location and edge. The experimental results show that this method can distinguish the edge and texture of breast tumors from the background, providing better edge and shape information for the second-stage classification network. The Attention-Unet model structure is shown in Figure 3. 

**Figure 4:**
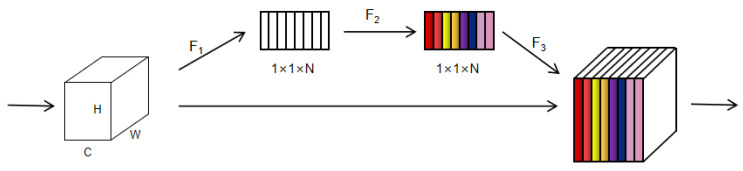
Attention module structure diagram. The feature graph is weighted by a 1 × 1 convolution.

The attention module integrated in the decoding layer can provide the neural network with the ability to focus on its output. The core’s function is to make the network pay more attention to the place to which it needs to pay attention, which is generally reflected by weight. The main implementation method is that the input and output are the current layer of the encoder and the decoder layer of the corresponding size, respectively. Firstly, the input is a C × H × W feature map, and the feature map of 1 × 1 × N is obtained using the activation function F1. The feature map matches the number of feature channels of the input and represents the global distribution of the feature response. Then, each feature channel is weighted by the activation function F2, which is used to represent the correlation and weight between feature channels. Finally, the activation function F3 is used to restore the feature map, and the previous features are weighted by channel to output the feature map with different weight information. In this way, the attention coefficient can be used to weight the feature map so as to obtain the final feature map. The attention module structure diagram is shown in Figure 4.

**Figure 5:**
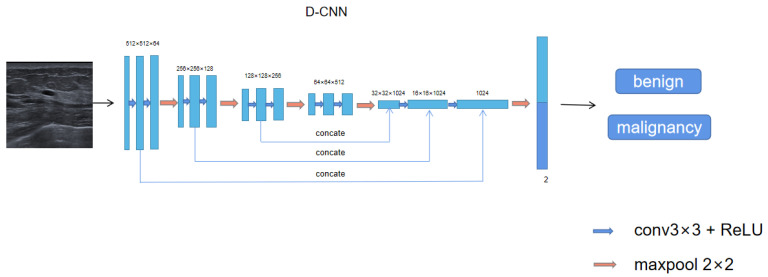
The D-CNN model consists of a feature extraction module and a fully connected layer for feature fusion. The model can extract 1 × 64 global feature maps from the complete original image. The lower model layer can reduce the dimension of the original feature map through the pooling layer. At the end of the CNN model, the global feature map and local feature map of the full connection layer are fused to output a two-dimensional vector and predict the optimal classification label of the input image.

Then, the mask result identified by the first network is superimposed with the original image to obtain the tumor coordinate position, and a 512 × 512 patch centered on the tumor region is simulated according to its position. For tumors in the edge region, 0 is added to avoid deformations in the tumor region, which is used as the training set of the second network classifier, D-CNN. Then, the classification model is trained by this deep convolutional neural network. The D-CNN network is connected through multi-layer convolution, pooling and full connection layers. resnet18 is used as the basic feature extraction layer, and multi-scale feature fusion technology is used in the middle layer to obtain the feature maps of the high-level network and the low-level network, respectively. Then, the high-dimensional features and low-dimensional features are fused through a concatenate operation to superposition multiple feature maps. Then, the multi-layer feature maps are integrated through the fully connected layer to predict the category with the highest score. The D-CNN model structure diagram is shown in Figure 5.

### 2.4. Evaluation Indicators

(1) MIoU and Dice are used to evaluate the first deep convolutional neural network.

MIoU: The MIoU is the average intersection ratio, and the formula is area of overlap/area of union, which mainly determines the intersection and union ratio of the real and predicted values.

Dice: The Dice coefficient is a common dichotomous index used to measure the degree of overlap between the predicted segmentation and true segmentation.

(2) The most commonly used indicators in a medical evaluation for the second-degree convolutional neural network include accuracy (ACC), sensitivity, specificity and the area under the curve. The sensitivity is also called the true positive rate (TPR), which is the probability that a patient is classified as malignant, and the specificity is called the true negative rate (TNR), which is the probability that a person who does not actually have the disease is classified as benign. The AUC is the area bounded by the axis under the receiver operator characteristic curve (ROC).

## 3. Result

### 3.1. Model Performance Evaluation

**Figure 6:**
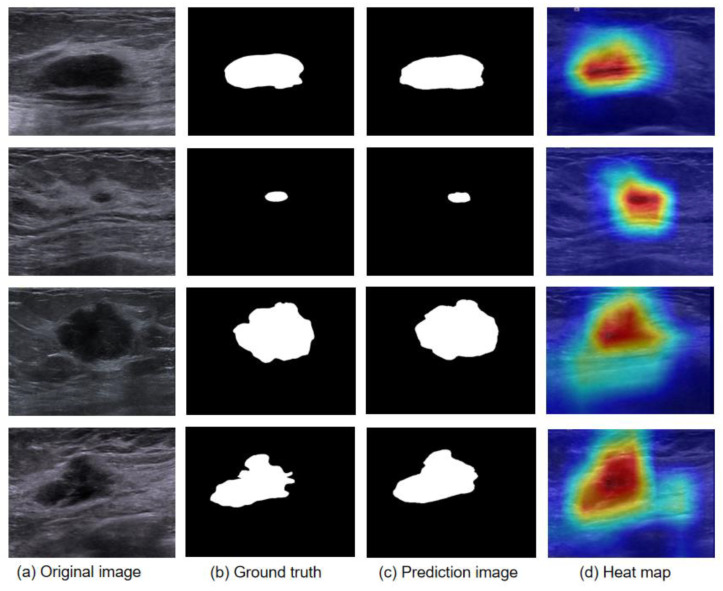
Segmentation model identification results show: (**a**) original image; (**b**) ground truth, The mask confirmed by the doctor; (**c**) prediction image, the mask identified by the model; (**d**) heat map, probabilistic heat map of model output. The closer the color is to red, the deeper the model’s focus.

We tested the validity of multiple models on breast ultrasound image datasets, and as a comparison, we also used a U-net network with no added attention mechanism to demonstrate the validity of the model’s added attention mechanism. The MIoU and Dice coefficients of the breast tumor localization module were about 0.89 and 0.92, respectively. As shown in Table 2, we compared the results of different network models in the verification of tumor region recognition on breast ultrasound images. A comparison of the final results shows that our model is superior to other methods in terms of the evaluation indexes. The comparison of model recognition results is shown in Figure 6. 

The experimental results clearly demonstrate that the Attention-Unet model we used plays a powerful role in the recognition of breast tumors. By comparison with the label map, the recognition results of the model show that it can distinguish the tumor and the background region, and the details of the edge region are also detailed. We also output the intermediate process of the model, and the probability heat map also proves that the focus position of the model is correct.

**Figure 7:**
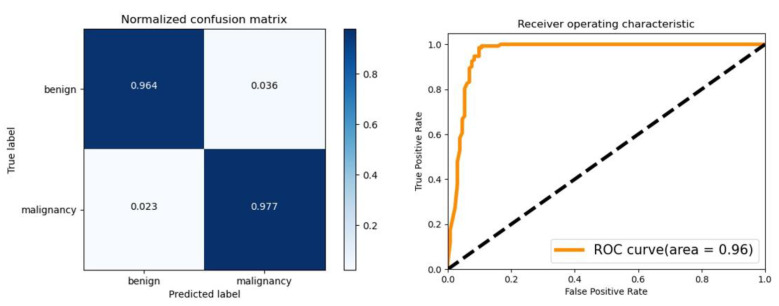
Confusion matrix and ROC curve. When tested using a dataset with pathological results, the confusion matrix of the diagnostic procedure is shown in the left figure, with a sensitivity of 97.7% and a specificity of 96.4% for diagnosing benign and malignant tumors. The ROC curve of the diagnostic is shown on the right, with an AUC of 0.96.

For the classification network, D-CNN, we evaluate its final performance using accuracy, sensitivity and specificity. The accuracy of the model is 97%, the sensitivity is 97.7%, and the specificity is 96.4%. The results show that our model can distinguish between benign and malignant breast tumors. The AUC and ROC curves are shown in Figure 7.

### 3.2. External Data Validation

**Figure 8:**
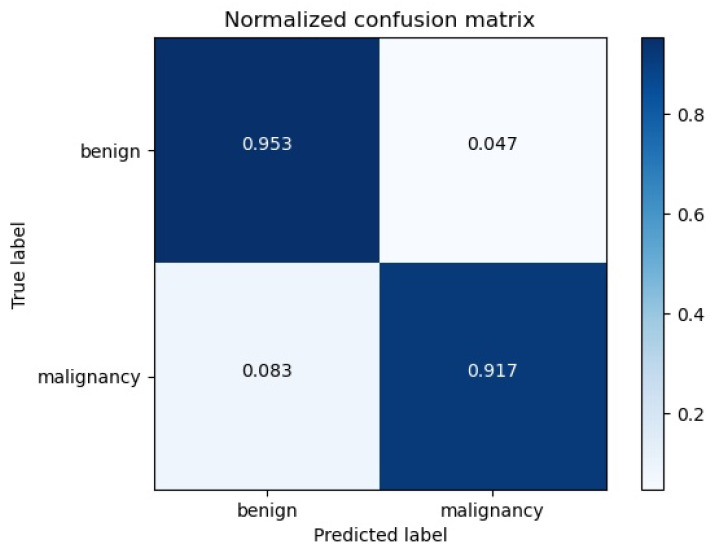
Confusion matrix. We tested the externally validated data and showed an accuracy of 94%, sensitivity of 91.7% and specificity of 95.3%.

To verify the generalization ability of our proposed algorithm, we collected data from 100 patients from Ejin Banner Hospital in Inner Mongolia as an external validation set. Each patient had a clear image of the tumor, and all of them were female patients from China. We used our model to test the data on various metrics, including accuracy, sensitivity and specificity. The results show that the accuracy of the model is 94%, the sensitivity is 91.7% and the specificity is 95.3%, showing the same excellent performance. However, compared with the original data set, the test results show that the model has potential clinical application, which also proves that our algorithm can achieve the same performance using the data of different hospitals and machines. The Confusion matrix of validation dataset is shown in Figure 8. 

### 3.3. Ablation Experiment

**Figure 9:**
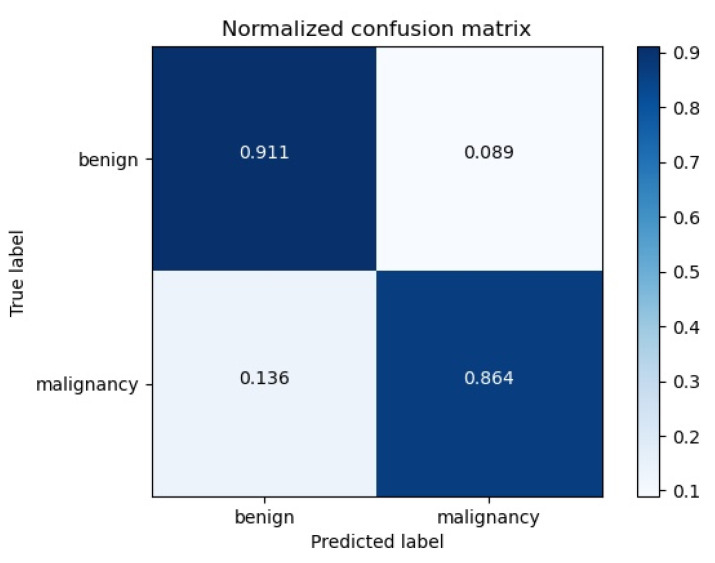
Confusion matrix. We tested the externally validated data and showed an accuracy of 89%, sensitivity of 86.4% and specificity of 91.5%.

In order to verify the validity of the two-stage network we used, we designed an ablation experiment to demonstrate that the segmented and unsegmented data have a certain impact on the classification results. Specifically, we designed the experiment using the same dataset and distribution as the unsegmented method and, in order to ensure the fairness of the experiment, we used the same hyperparameters to ensure optimal performance and a fair evaluation. The confusion matrix without the segmentation algorithm is shown in Figure 9.

The different methods used in the two datasets are shown in the Table 3:

The experimental results show that the model index trained by the unsegmented tumor data is lower than that trained after data segmentation. Due to the complexity of ultrasound images, the background region has a great impact on the judgment of the model, and the two-stage model we propose can avoid this problem, which also verifies the rationality of our design algorithm.

### 3.4. Design of Intelligent Auxiliary Diagnosis System for Breast Ultrasound Imaging

In order to make the artificial intelligence algorithm more interactive, we designed and developed an intelligent assisted-diagnosis system for breast ultrasound. The main object of this system is the clinician. Through end-to-end recognition, the sonographer only needs to input the acquired images into our system and can immediately obtain the location of the tumor and the benign and malignant results.

**Figure 10:**
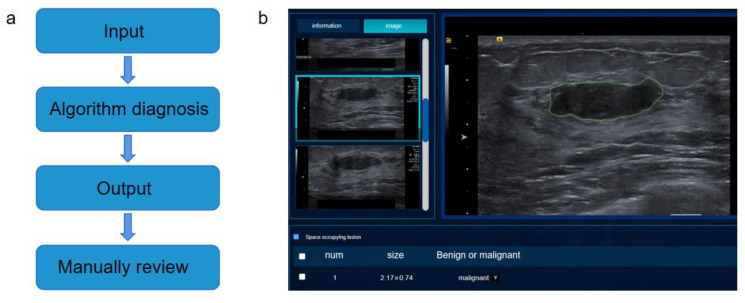
(**a**) Diagnostic system logical structure diagram. The diagnostic system consists of 4 steps: (1) image upload, in which the doctor uploads the image that needs to be detected to the diagnostic system; (2) AI analysis, using the algorithm model to identify the location and category of breast tumors; (3) output results and visualization, displayed in the front-end interface; and (4) the doctor evaluates the results and judges the correctness of the output. (**b**) Diagnostic system interface. The interface of the diagnostic system includes the information area and the image area, and the information area includes the number of breast tumors, tumor size and whether the tumor is benign or malignant. The image area contains the results and position outline of the recognition image.

The system design is mainly divided into several steps: 1. Upload pictures: the ultrasound images taken by the doctor, including JPG, PNG and DICOM formats, are uploaded directly to the system. 2. Through AI analysis, the location and type of breast tumor nodules, benign or malignant, are identified by using an algorithm, and the results are displayed on the software. 3. Doctor discrimination, where the accuracy of system output is tested through the recognition results of the doctor’s judgment algorithm. The software diagnosis logic and interface are as follows Figure 10.

### 3.5. Clinical Trial Design

Ultrasound doctors with different years of experience may not make the same judgment regarding tumor images. In order to verify the effectiveness of our algorithm in clinical diagnosis for sonographers, we designed a multi-viewer, multi-data sample validation test. We invited three sonographers of different seniorities as the study group, with more than 20 years, 3 years and 1 year of ultrasonography experience, respectively. We divided the three sonographers into three independent groups, and they acquired completely independent test results. The test process was divided into two stages.

In the first stage, we first let the senior and two junior readers interpret the ultrasonic images directly, without using the algorithm results. In the second stage, after a washout period, our algorithm gave the result as a reference, and then the viewer carried out a new round of interpretation.

In this experiment, the film readers used the algorithm-assisted interpretation results as the experimental group, the interpretation results without algorithm-assisted diagnosis as the control group and the pathological results as the fine standard. Based on tumor location accuracy, benign and malignant accuracy and the time required to diagnose 100 patients, we finally came to the following conclusions. The Results of clinical trial are shown in the Table 4 and Table 5:

As can be seen from the above table, the benign and malignant diagnosis of breast tumors by senior doctors in the control group and the experimental group was not affected, but the time to diagnosis was more than doubled, mainly because we could mark the tumor location, reducing the interpretation of tumor location by senior doctors and improving the diagnostic efficiency. For junior doctors, the results of the trial group show that our approach can significantly reduce the time it takes to locate tumors in complex ultrasound images, and the labeled locations and results can also assist junior doctors and improve their judgement accuracy.

## 4. Conclusions

In this paper, we introduce a multi-task framework combining tumor segmentation and classification tasks in breast ultrasound images. Our proposed method mainly consists of two modules: a U-net segmentation module based on attention mechanism and a D-CNN classification model. In order to enhance the extraction of spatial position information in the image, we used the attention mechanism to increase the position weight of features to improve the segmentation accuracy. Moreover, the classification of benign and malignant tumors by using segmentation images also confirmed that the preservation of local tumor regions was superior to the classification of the whole image. The experimental results on multi-center datasets show that our method has good generalization performance. Compared with other state-of-the-art methods, our method is capable of end-to-end identification, taking into account both performance and timeliness, and can help sonographers improve diagnostic efficiency in clinical practice.

## 5. Discussion

Although our experimental results have demonstrated the validity of our proposed model, there are some limitations. First of all, most ultrasound images in clinical practice are continuous DICOM images, and benign and malignant tumors with different frame numbers and angles are not necessarily the same. However, we used 2D datasets, ignoring the correlation information between different layers. Secondly, the patients in the dataset we used were all from the same region, and whether there are differences between different ethnic groups remains to be studied. Finally, although our model shows some results in simulated scenarios, its performance in real medical settings needs to be further explored.

To address these limitations, we plan to develop better models, such as data in a DICOM format, that incorporate continuity information between the different layers of the image. In addition, we would like to experiment with more center data and doctors using our auxiliary diagnostic software, which will further validate the practicability of our method in clinical medical diagnosis and contribute to more efficient and accurate breast tumor identification in the future.

## Figures and Tables

**Table 1:** The number of breast tumor images for the training, validation and test.

Diagnosis	The Images Used as Training and Validation Datasets		The Images Used as Test Datasets	
	Number of Patients (n)	Number of Images (n)	Number of Patients (n)	Number of Images (n)
benign	649	1288	56	132
malignancy	482	769	44	132

**Table 2:** Performance evaluation of segmentation network model.

Model	MIoU	Mdice
U-net	0.82	0.84
Fast-RCNN	0.83	0.85
Deeplab V3	0.85	0.87
Ours	0.89	0.92

**Table 3:** Comparison of classification performance before and after segmentation.

Model	Accuracy	Sensitivity	Specificity	AUC
U-net + D-CNN	97%	97.7%	96.4%	0.96
D-CNN	89%	86.4%	91.5%	0.87

**Table 4:** Results for control group.

Medical Seniority	Accuracy of Benign and Malignant	Time
1 year	71%	60 min
3 years	83%	45 min
20 years	98%	42 min

**Table 5:** Results for trial group.

Medical Seniority	Accuracy of Benign and Malignant	Time
1 year	85%	40 min
3 years	92%	34 min
20 years	98%	16 min

## Data Availability

Data are not available at present because there are further research plans. If necessary, you can contact the author to obtain the data.
